# Fluid Intelligence in Children with Learning Disabilities

**DOI:** 10.11621/pir.2025.0102

**Published:** 2025-03-01

**Authors:** Irina E. Rzhanova, Olga S. Alekseeva, Viktoriya S. Britova, Yulia A. Burdukova

**Affiliations:** a Federal Scientific Center for Psychological and Interdisciplinary Research, Moscow, Russia; b Moscow State University of Psychology and Education, Russia

**Keywords:** fluid intelligence, learning disabilities, children, WISC-V, KABC-II

## Abstract

**Background:**

Fluid intelligence is an integral cognitive ability that involves solving new non-standard problems. It strongly predicts academic and professional achievement, whereas a low level of fluid intelligence is an important predictor of learning problems. Clinical studies of fluid intelligence are of interest for the development of training programs in various groups of children with special needs. This article presents a study on fluid intelligence in children with learning disabilities.

**Objective:**

This study aimed to investigate characteristics of fluid intelligence and its relationships with other cognitive characteristics in children with learning disabilities.

**Design:**

This study involved 93 children, divided into two groups: 55 typically developing children (control group) and 38 children with learning disabilities (clinical group). To assess intelligence characteristics, this study employed the Kaufman Assessment Battery for Children (KABC-II) and the Wechsler Intelligence Scale for Children Fifth Edition (WISC-V).

**Results:**

A reduction was found in fluid intelligence, working memory, short-term memory, long-term memory, processing speed, visual-spatial abilities, and verbal abilities in the group of children with learning disabilities compared to the control group. In the clinical group, fluid intelligence was strongly associated with a greater number of cognitive parameters compared to the control group.

**Conclusion:**

It is possible to assume that a close connection of fluid intelligence with the assessed cognitive characteristics in the group of children with learning disabilities may be due to general challenges in cognitive development.

## Introduction

Fluid intelligence is a complex cognitive ability that provides thought processes with the flexibility to solve new and non-standard problems (McGrew, 2009; Schneider, McGrew, 2012). It is determined mainly by innate factors and depends little on the cultural experience of the individual, while at the same time, it largely determines the speed and efficiency of acquiring knowledge and skills. This ability is an important element of cognitive development in general, ensuring children’s acquisition of new cognitive skills and abilities (Cattell, 1987; Blair, 2006; Green et al., 2017). As an integral characteristic, fluid intelligence is involved in all cognitive processes and is considered one of the most important factors of learning. It has been found that it predicts academic and professional success, especially in tasks involving intellectual work (Alekseeva et al., 2021; Lynn et al., 2007; Otero, 2017). On the other hand, a low level of fluid intelligence is an important predictor of learning problems (Lynn et al., 2007; Nisbett, 2009).

In the most widely recognized theory of intelligence, the Cattell–Horn–Carroll hierarchical model, fluid intelligence is defined as the ability to approach current problems flexibly and adaptively which cannot be solved solely through previously learned schemes and algorithms (McGrew, 2009). This complex construct is primarily intended for finding solutions in unfamiliar, non-standard situations but is also involved in everyday, routine tasks when existing knowledge and skills are insufficient. Fluid intelligence is engaged in constructing logical reasoning, forming concepts and representations, classifying unfamiliar stimuli, constructing and testing hypotheses, identifying significant features of objects and phenomena, determining their properties, differences, and connections, comprehending new knowledge, and making inferences based on it, and making justified assumptions in uncertain situations (Schneider, McGrew, 2012).

Fluid intelligence consists of three specific abilities: induction, general sequential reasoning, and quantitative reasoning. Induction is the ability to explore phenomena and situations and to identify underlying principles and patterns. General sequential reasoning, sometimes also referred to as deduction, is defined as the capacity to utilize known principles and patterns for reasoning and problem-solving. Quantitative reasoning involves the ability to apply induction or general sequential reasoning to discern quantitative relationships and perform mathematical operations. Modern research methods in fluid intelligence are geared towards assessing these abilities (Schneider, McGrew, 2012).

The relationship between fluid intelligence and other cognitive characteristics continues to generate interest among scientists. The findings from existing research are quite contradictory, which is partly due to the theoretical problem of delineating the constructs under investigation. Additionally, the use of a wide range of diagnostic methods, not all of which align with current understandings of fluid intelligence, contributes to these inconsistencies.

Currently, there is consensus on the existence of a strong relationship between fluid intelligence and working memory (Brydges et al., 2021; Schroeders et al., 2016; Conway et al., 2002; Cowan et al., 2005). Nearly all studies that explore ways to enhance fluid intelligence suggest training specifically targeting working memory (Jaeggi et al., 2011; Rzhanova et al., 2020). However, the issue of identifying possible mediators of the connection between fluid intelligence and working memory remains topical. Most frequently, cognitive characteristics such as short-term memory (Tillman et al., 2008; Hornung et al., 2011; Colom et al, 2006) and attention control (Engle, 2010; Schroeders et al., 2016;) are considered as mediators.

Researchers studying fluid intelligence also focus on other related cognitive characteristics, particularly visual-spatial and verbal abilities. A close interconnection between visual-spatial abilities and fluid intelligence has been demonstrated, despite some diagnostic challenges in distinguishing these cognitive constructs (Gizzonio et al., 2022). In cognitive research, verbal abilities are typically considered a component of crystallized intelligence, and their close relationship with fluid intelligence is attributed to the contribution of fluid intelligence to the formation of crystallized intelligence (Thorsen et al., 2014; Carpentier et al., 2022).

Clinical research findings are of particular interest in the study of fluid intelligence, providing material for a deeper understanding of cognitive impairments in various clinical groups. Such studies open new avenues for corrective interventions with children who have developmental peculiarities, as well as with adults whose cognitive functions have been impacted by various life events. These insights are crucial for developing tailored therapeutic strategies that can potentially improve or mitigate the cognitive deficits observed in these populations.

Fluid intelligence and its connections with other cognitive abilities have been extensively studied across various clinical groups, including individuals with Attention Deficit Hyperactivity Disorder (ADHD), Autism Spectrum Disorder (ASD), consequences of traumatic brain injuries, preterm infants, and others (Morgan et al., 2019; Brydges et al., 2021; Tamm, Juranek, 2012). According to psychometric data, the fluid intelligence index is significantly lower compared to control groups in children with developmental delays and in children with established risk factors for developmental issues, which include low birth weight and asphyxia at birth (Wechsler, 2014).

It should be noted that children and adults with Attention Deficit Hyperactivity Disorder (ADHD) are of particular interest in the context of studying fluid intelligence (Tamm, Juranek, 2012; Morgan et al., 2019; Brydges et al., 2021). This focus is primarily due to the fact that ADHD fundamentally involves impairments in executive functions (such as attention and working memory), which are closely linked to fluid intelligence (Schroeders et al., 2016; Conway et al., 2002; Cowan et al., 2005). The use of neuroimaging techniques during the performance of various cognitive tasks has revealed reduced activation in brain areas responsible for fluid thinking in children with attention deficits (Tamm, Juranek, 2012).

There is a well-founded assumption that in children with learning disabilities, fluid intelligence plays a leading role in the formation of difficulties in mastering new skills (Blair, 2004). Studies of the relationship between fluid intelligence and learning disabilities have demonstrated a significant influence of fluid intelligence on academic performance in reading and mathematics in elementary grades, with a gradual decrease in this influence with age (Evans et al., 2001).

Despite the broad interest in researching fluid intelligence and its impact on cognitive development and educational success, relatively few studies have been conducted with subjects who have learning disabilities. The availability of data suggesting the potential for improving fluid intelligence through specifically designed training sessions makes the investigation of this construct in children with developmental delays particularly relevant.

The aim of this study is to explore the characteristics of fluid intelligence and its relationships with other cognitive characteristics in children of primary school age with learning disabilities. During the course of this research, the following questions were posed:

1. How do the cognitive indicators of children with learning disabilities differ from those of typically developing children?
2. What contribution does the measure of fluid intelligence make to the structure of cognitive functions in the two groups of children?

## Methods

### Participants

This study involved 93 children, divided into two groups: 55 typically developing children and 38 children with learning disabilities. The control group consisted of pupils from the 2nd to 4th grades of a general education school, comprising 27 boys and 28 girls. The age range was from 96 to 132 months, with an average age of 115.8 months and a standard deviation of 9.7 months.

The group of children with learning disabilities was recruited from pupils in the 1st to 4th grades of a special education school that follows the Federal State Educational Standard of the seventh type; prior to admission to this school, all children undergo a psychological/medical/pedagogical assessment, and each child’s personal file contains a confirmed diagnosis of learning disabilities. This group comprised 22 boys and 16 girls. The age at the time of this study ranged from 97 to 144 months, with an average age of 124 months and a standard deviation of 13.6 months.

### Procedure

To assess fluid intelligence, this study employed scales from the *Kaufman Assessment Battery for Children (KABC-II)* (Kaufman, Kaufman, 2004) and the *Wechsler Intelligence Scale for Children Fifth Edition (WISC-V)* (Wechsler, 2014). Other integral cognitive indicators included in these tests were also diagnosed.

*The KABC-II* test not only diagnoses the overall intelligence quotient but also provides assessments across four scales:

1. Short-term memory (Gsm); subtests include Number Recall and Word Order.
2. Visual processing (Gv); subtests include Rover and Triangles.
3. Fluid intelligence (Gf); subtests include Story Completion and Pattern Reasoning.
4. Long-term memory (Glr); subtests include Atlantis and Rebus.

*The WISC-V* test is designed to diagnose overall intelligence and five integral indicators:

1. Verbal Comprehension Index (VCI); subtests include Similarities and Vocabulary.
2. Visual Spatial Index (VSI); subtests include Block Design and Visual Puzzles.
3. Fluid Reasoning Index (FRI); subtests include Matrix Reasoning and Figure Weights, Picture Concepts, and Arithmetic.
4. Working Memory Index (WMI); subtests include Digit Span and Picture Span.
5. Processing Speed Index (PSI); subtests include Coding and Symbol Search.

In processing the collected data, both an analysis of variance (ANOVA) and a regression analysis were used. A statistical analysis was conducted using the SPSS software, version 19.

## Results

### Differences in Cognitive Abilities

**Figure 1:**
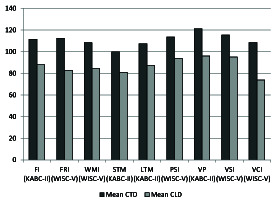
Differences in cognitive indices between children with learning disabilities and typically developing children

*Notes. CTD—children with typical development; CLD—children with learning disabilities; FI—Fluid Intelligence; FRI—Fluid Reasoning Index; WMI—Working Memory Index; STM—Short-Term Memory; LTM—Long-Term Memory; PSI—Processing Speed Index; VP—Visual Processing; VSI—Visual Spatial Index; VCI—Visual Comprehension Index.*

In the initial stage of this study, a comparison of cognitive characteristics was conducted between children of primary school age with learning disabilities and their typically developing peers. A one-way Analysis of Variance (ANOVA) was used to assess these differences. The results are presented in *Figure 1*.

The calculated F-values clearly demonstrate significant differences across all assessed cognitive parameters, indicating that children with learning disabilities scored lower on the respective tests compared to their typically developing peers. Specifically, primary school-aged children from the study group exhibited poorer performance on tasks assessing fluid intelligence, working memory, short-term and longterm memory, processing speed, visual-spatial abilities, and verbal abilities.

### Contribution of Fluid Intelligence to Cognitive Performance

To assess the contribution of fluid intelligence to the cognitive characteristics being studied, a regression analysis was performed. Significant predictors were selected separately for the subtests and scales from three initial sets of variables: the subtests of WISC-V, the subtests of KABC-II, and the primary scales from both the WISC-V and KABC-II. The selection method used was stepwise regression. The analysis in this work presents regression models that explain the largest contribution in variance of the dependent variables.

The results of the regression models, with working memory as the dependent variable, are presented in *Tables 1 and 2*.

**Table 1:** Regression models for the dependent variable “Working Memory” (children with typical development)

	R^2^	F	β	p
**Model 1**	.450	11.978		.000
Short-term memory			.436	.000
Processing Speed Index			.359	.003
Fluid intelligence (KABC-II)			.245	.041
**Model 2**	.505	14.989		.000
Similarities			.439	.000
Symbol Search			.291	.012
Word Order			.263	.022

**Table 2:** Regression models for the dependent variable “Working Memory” (children with learning disabilities)

	R^2^	F	β	p
**Model 1**	.687	22.667		.000
Short-term memory			.468	.000
Visual processing			.321	.016
Fluid intelligence (KABC-II)			.308	.026
**Model 2**	.745	21.964		.000
Number Recall			.502	.000
Triangles			.334	.004
Triangles			.328	.011
Picture Concepts			.234	.039

According to the data presented, fluid intelligence is a significant predictor of working memory in both groups of subjects. In the group of typically developing children, the integral scale of fluid intelligence from KABC-II is one of three predictors of working memory; the other predictors include scales for short-term memory and processing speed. In the group of children with learning disabilities, the integral scale of fluid intelligence from KABC-II also appears as one of three predictors of working memory, alongside scales for short-term memory and visual-spatial abilities.

The regression models constructed from data obtained in the group of typically developing primary school children explain 45 and 51 of the variance in working memory when predictors are selected from the scales and subtests, respectively. For the group of primary school children with learning disabilities, the regression models, compiled in a similar manner, account for 69 and 75 of the variance in working memory.

The results of the regression analysis, with long-term memory as the dependent variable, are presented in *Tables 3 and 4*.

**Table 3:** Regression models for the dependent variable Long-Term Memory (children with typical development)

	R^2^	F	β	p
**Model 1**	.240	14.488		.000
Verbal Comprehension Index			.489	.000
**Model 2**	.307	9.961		.000
Vocabulary			.454	.001
Number Recall			.268	.037

**Table 4:** Regression models for the dependent variable Long-Term Memory (children with learning disabilities)

	R^2^	F	β	p
**Model 1**	.215	9.031		.005
Fluid intelligence (KABC-II)			.464	.005
**Model 2**	.182	7.326		.011
Pattern Reasoning			.426	.011

In the group of typically developing children, verbal abilities are the primary predictor of long-term memory. When selecting independent variables from the scales, the Verbal Comprehension Index verbal index is the only significant predictor of long-term memory. When selecting independent variables from the subtests, in addition to the Vocabulary subtest, which is part of the Verbal Comprehension Index, the Number Recall subtest from KABC-II, which assesses short-term memory, also emerged as a significant predictor.

In the group of children with learning disabilities, the only significant predictors of long-term memory, when selecting independent variables from both the scales and the subtests, are the measures of fluid intelligence. Specifically, the integral scale of fluid intelligence from KABC-II and the Pattern Reasoning subtest, which is part of this scale, stand out. Clearly, it is possible to discuss the influence of fluid intelligence on long-term memory only in the group of primary school children with learning disabilities.

The results of the regression analysis, where the information processing speed index was considered as the dependent variable, are presented in *Tables 5 and 6*. In the group of typically developing children, working memory is the only significant predictor of information processing speed, explaining 19 of its variance. In the group of children with learning disabilities, fluid intelligence is the only predictor of processing speed, accounting for 24 of the variance of the dependent variable.

**Table 5:** Regression models for the dependent Variable Processing Speed (children with typical development)

	R^2^	F	β	p
**Model 1**	.194	12.268		.001
Working Memory Index			.440	.001
**Model 2**	.180	11.232		.002
Picture Span			.425	.002

**Table 6:** Regression models for the dependent Variable Processing Speed (children with learning disabilities)

	R^2^	F	β	p
**Model 1**	.239	11.296		.002
Fluid intelligence (KABC-II)			.489	.002
**Model 2**	.252	11.454		.002
Story Completion			.502	.002

The results of the regression analysis for the Visual-Spatial index are presented in *Tables 7 and 8*. In both groups of subjects, fluid intelligence is the only significant predictor of visual-spatial abilities. In the group of children with learning disabilities, fluid intelligence accounts for 44 of the explained variance of the dependent variable, while in the control group, it accounts for 54%.

**Table 7:** Regression models for the dependent variable Visual Spatial Index (children with typical development)

	R^2^	F	β	p
**Model 1**	.394	33.151		.000
Fluid Reasoning Index			.628	.000
**Model 2**	.539	29.185		.000
Arithmetic			.509	.000
Matrix Reasoning			.373	.001

**Table 8:** Regression models for the dependent variable Visual Spatial Index (children with learning disabilities)

	R^2^	F	β	p
**Model 1**	.340	18.530		.000
Fluid Reasoning Index			.583	.000
**Model 2**	.442	28.465		.000
Matrix Reasoning			.664	.000

The results of the regression analysis, where the verbal abilities index was considered as the dependent variable, are presented in *Tables 9 and 10*. In the group of typically developing children, fluid intelligence and long-term memory serve as predictors of verbal abilities when selecting independent variables from both the scales and subtests. The largest proportion of explained variance for the dependent variable is 48%. In the group of children with learning disabilities, fluid intelligence is the sole predictor of verbal abilities, with the largest proportion of variance explained by it being 21%.

**Table 9:** Regression models for the dependent variable Verbal Comprehension Index (children with typical development)

	R^2^	F	β	p
**Model 1**	.430	16.964		.000
Fluid Reasoning Index			.445	.000
Long-term memory			.402	.001
**Model 2**	.481	9.972		.000
Arithmetic			.323	.009
Rebus			.279	.018
Story Completion			.278	.019
Picture Concepts			.254	.033

**Table 10:** Regression models for the dependent variable Verbal Comprehension Index (children with learning disabilities)

	R^2^	F	β	p
**Model 1**	.193	8.586		.006
Fluid Reasoning Index			.439	.006
**Model 2**	.210	9.586		.004
Fluid Reasoning Index			.459	.004

Thus, fluid intelligence emerges as a predictor of verbal abilities in both groups studied. Similar to the case with visual-spatial abilities, it is not possible to say whether its contribution to the dependent variable is greater or less in one group compared to the other.

Thus, the conducted regression analysis demonstrated the influence of fluid intelligence on working memory, visual-spatial abilities, and verbal abilities in the group of typically developing children. In the group of children with learning disabilities, fluid intelligence is associated with a broader range of cognitive parameters and serves as a predictor of working memory, long-term memory, processing speed, visual-spatial abilities, and verbal abilities; the contribution of fluid intelligence to short-term memory is appropriately considered to be mediated through working memory. Moreover, the impact of fluid intelligence on working memory is notably stronger in the group of children with learning disabilities than in the group of typically developing children.

## Discussion

During this study, an analysis was conducted on the characteristics of fluid intelligence and its associations with working memory, short-term memory, long-term memory, processing speed, visual-spatial abilities, and verbal abilities in children with typical development and those with learning disabilities. Using a one-way ANOVA, a comparison was made between the clinical and control groups. Significant differences were found across all studied indicators between the two groups of primary school children, with the statistical significance of these differences being quite high. It can be confldently stated that there is a consistent reduction in fluid intelligence, working memory, short-term memory, long-term memory, processing speed, visualspatial abilities, and verbal abilities in the group of children with learning disabilities compared to the control group. Most studies of children with learning disabilities have shown significant impairments in working memory and information processing speed compared to typically developing children (Cornoldi et al., 2014; Giofrè, Cornoldi, 2015). In our study, significant differences were found in all cognitive indices. This may be due to the specifics of the sample and the school system in Russia. Schools that educate children with learning disabilities generally recruit children who, in addition to specific academic difficulties, are at the lower limit of the intellectual norm. They can be classified as possessing borderline intellectual functioning. This term was used some time ago but was excluded from the DSM, since strictly speaking, it is still an intellectual norm. However, there is a well-founded opinion on the need to reintroduce this term into scientific and practical use (Wieland, Zitman, 2016).

The vast majority of cases of learning disabilities are associated with minimal cerebral/organic damage to the brain (Emelina, Makarov, 2018). Fluid intelligence is primarily determined by innate structural and functional features of the brain and is minimally influenced by cultural factors (Cattell, 1963; McGrew, 2009); its decline suggests lag in the development of other cognitive indicators, primarily due to its crucial role in the formation of overall intelligence during ontogeny.

To assess the connections between fluid intelligence and other cognitive abilities and clarify its contribution to cognitive development, a regression analysis was conducted. Fluid intelligence is a significant predictor of working memory in both groups of subjects. Along with fluid intelligence, short-term memory and visualspatial abilities contribute to working memory in the clinical group, while in the control group, short-term memory and processing speed also play roles. Notably, the connections between fluid intelligence and working memory are significantly stronger in children with learning disabilities. This finding aligns with the literature. Theories on the development of fluid intelligence and working memory in ontogeny, which suggest a non-linear relationship between age and development pace (Detterman, Daniel, 1989; Fry, Hale, 1996; Siegel, 1994), and the activation of similar brain cortex areas during tasks involving fluid intelligence and working memory (Chai et al., 2018) explain these results. We believe that cognitive delays related to organic CNS damage result in a stronger connection between fluid intelligence and working memory in children with diagnosed learning disabilities compared to their typically developing peers.

Our investigation into the contribution of fluid intelligence to long-term memory showed that in the clinical group, fluid intelligence is the only significant predictor of long-term memory. In the control group, verbal abilities serve as the primary predictor of long-term memory, with short-term memory being less significant and only when selecting predictors from among the subtests. The results obtained in the group of children with learning disabilities are consistent with the notion that long-term memory is a characteristic independently associated with fluid intelligence (Mogle et al., 2008). The principle of the inverse relationship between general intelligence and the strength of connections among cognitive ability indicators, identified by D. Detterman and M. Daniel, may explain the discrepancies observed between the clinical and control groups (Detterman, Daniel, 1989). This investigation into the contribution of fluid intelligence to processing speed revealed that fluid intelligence is a significant predictor of information processing speed only in the clinical group. In the control group, working memory serves as the predictor of processing speed.

The results of the regression analysis demonstrate a strong relationship between fluid intelligence and visual-spatial abilities, aligning with data presented in other studies (Gizzonio et al., 2022; Colom et al., 2009). Fluid intelligence significantly contributes to visual-spatial abilities in both children with typical development and those with learning disabilities. Fluid intelligence is also a significant predictor of verbal abilities in both groups of subjects, and it serves as the sole significant predictor in children with learning disabilities. Thus, the results of the regression analysis indicate that in children with learning disabilities, fluid intelligence is associated with a greater number of cognitive parameters compared to typically developing children.

## Conclusions

A reduction was found in all measured cognitive parameters in the group of children with learning disabilities compared to the group of children with typical development. In the clinical group, fluid intelligence is strongly associated with a greater number of cognitive parameters compared to the control group. It is possible to assume that a close connection of fluid intelligence with the assessed cognitive characteristics in the group of children with learning disabilities may be due to general challenges in cognitive development.

## Limitations

The limitations of this study are related to the available data. Although the children participating in this study lived in the same language region, we collected no data on the socio-demographic status of their families; therefore, it is not possible to conclude that the clinical and control groups were equal for this parameter.
